# Linking reward processing to behavioral output: motor and motivational integration in the primate subthalamic nucleus

**DOI:** 10.3389/fncom.2013.00175

**Published:** 2013-12-17

**Authors:** Juan-Francisco Espinosa-Parrilla, Christelle Baunez, Paul Apicella

**Affiliations:** Institut de Neurosciences de la Timone, CNRS-Aix-Marseille UniversitéMarseille, France

**Keywords:** basal ganglia, reinforcement, reward expectation, context processing, single-neuron activity, monkey

## Abstract

The expectation and detection of motivationally relevant events is a major determinant of goal-directed behavior and there is a strong interest in the contribution of basal ganglia in the integration of motivational processes into behavioral output. Recent research has focused on the role of the subthalamic nucleus (STN) in the motivational control of action, but it remains to be determined how information about reward is encoded in this nucleus. We recorded the activity of single neurons in the STN of two behaving monkeys to examine whether activity was influenced by the delivery of reward in an instrumental task, a Pavlovian stimulus-reward association, or outside of a task context. We confirmed preliminary findings indicating that STN neurons were sensitive not only to rewards obtained during task performance, but also to the expectation of reward when its delivery was delayed in time. Most of the modulations at the onset of reaching movement were combined with modulations following reward delivery, suggesting the convergence of signals related to the animal’s movement and its outcome in the same neurons. Some neurons were also influenced by the visuomotor contingencies of the task, i.e., target location and/or movement direction. In addition, modulations were observed under conditions where reward delivery was not contingent on an instrumental response, even in the absence of a reward predictive cue. Taken as a whole, these results demonstrate a potential contribution of the STN to motivational control of behavior in the non-human primate, although problems in distinguishing neuronal signals related to reward from those related to motor behavior should be considered. Characterizing the specificity of reward processing in the STN remains challenging and could have important implications for understanding the influence of this key component of basal ganglia circuitry on emotional and motivated behaviors under normal and pathological conditions.

## Introduction

Although it is traditionally considered that the subthalamic nucleus (STN) is important in motor control, an increasing number of studies has been conducted to investigate the role of this nucleus in the processing of reward-related information. Evidence in favor of the STN involvement in motivational processes comes primarily from lesions studies in behaving rats showing that STN dysfunction leads to increased responding for a food reward (Baunez et al., 2002). In addition, STN lesions could have a differential impact on the incentive motivational properties of natural reinforcers and drugs of abuse, the STN-lesioned rats becoming more motivated as they work to obtain food reward and less motivated when cocaine was used as the reward (Baunez et al., 2005), suggesting that processing of different types of rewards can be dissociated at the STN level.

Clinical studies also support the notion that the STN is a component of reward circuitry. In particular, deep brain stimulation (DBS) of this nucleus, which is effective at alleviating motor symptoms in patients with Parkinson’s disease, can also interfere with brain circuits that mediate mood and reward signals leading to enhanced motivation and decreased apathy in some of these patients (Funkiewiez et al., 2003; Takeshita et al., 2005), although an interference with decreased dopaminergic medication cannot be excluded. It has been also reported that DBS of the STN can either increase (Houeto et al., 2002; Schüpbach et al., 2005) or decrease (Witjas et al., 2005; Lhommée et al., 2012; Eusebio et al., 2013) the addiction of parkinsonian patients for their levodopa treatment. Since abnormal repetitive behaviors (i.e., compulsions) include an emotional component, the observation that obsessive–compulsive disorders can be improved by DBS of the STN is also an argument in favor of the contribution of this nucleus in emotional and motivational processes (Mallet et al., 2002; Baunez et al., 2011). Taking into account those elements, it has been suggested that the STN may represent a promising target for the treatment of addiction (Pelloux and Baunez, 2013).

Although, functional neuroimaging research in humans poses challenges to the interpretation of changes in activity of small subcortical brain structures (Keuken et al., 2013; reviewed in Péron et al., 2013), studies in this field have recently confirmed the role of the STN in behavioral inhibition highlighted by animal studies, such as the ability to cancel planned or already initiated actions (Aron and Poldrack, 2006; Li et al., 2008). While the role of this nucleus in motivation has received much less attention, the contribution of other parts of the basal ganglia, particularly the ventral striatum, in emotion and reward processes has been studied in many experiments using monetary or taste rewards under a variety of behavioral paradigms (Delgado, 2007). So far, hemodynamic changes restricted to the STN area which may be linked to anticipation and experience of reward have not been reported. However, as mentioned above, such an approach is still limited by the relatively poor spatial resolution of brain imaging techniques. On the other hand, neuronal recordings from the STN in patients with Parkinson’s disease or obsessive–compulsive disorders during DBS surgery or after electrode implantation (local field potentials, LFPs) have provided evidence that the STN region is active during the processing of information related to emotional aspects of behavior (Kühn et al., 2005; Brücke et al., 2007; Burbaud et al., 2013). A potential disturbance of emotional information processing at the STN level could account for the mood changes reported in parkinsonian patients subjected to STN stimulation (Krack et al., 2001; Schneider et al., 2003).

More direct evidence for the involvement of STN in motivational processes is obtained from single-neuron recording experiments in animals performing controlled behavioral tasks. Several components of the basal ganglia circuitry, including the striatum, globus pallidus and substantia nigra pars reticulata, have been implicated in the processing of reward-related information and in linking motivation to action (reviewed in Schultz et al., 2000 and Hikosaka et al., 2006). In contrast, there are few data in direct support of reward processing at the STN level. Previous studies in behaving rats have reported that STN neurons can be modulated after the presentation of stimuli associated with reward and at the time of the reward delivery (Teagarden and Rebec, 2007; Lardeux et al., 2009, 2013). Moreover, STN neurons show differential responses to reward-related cues according to the quality of the expected reward, i.e., the degree of sweetness of a sucrose solution (Lardeux et al., 2009), and they can also discriminate between food and drug reinforcement, showing a specialization according to the relative preference for the reward (Lardeux et al., 2013). These findings are consistent with the existing literature on the impact of STN inactivation in rodents, supporting a role for this nucleus in reward processing. Conversely, much less information about the role of the STN in motivation is available in the primate. In a preliminary report, we have examined the relation of STN neuronal activity to movement and reward in one monkey performing an arm reaching task (Darbaky et al., 2005). In that study, we showed that the discharge of STN neurons was often modulated during the movement period of the task and at the time of the reward delivery, suggesting that STN neurons carrying signals related to motor activity could also be informed about the reception of the reinforcer. However, the details as to how the association of reward information with motor processes occurs in this nucleus remain unclear.

The purpose of our study was to further examine in monkeys the activity of STN neurons which could be related to reward delivered in distinct behavioral situations. The activity was analyzed during performance of visually-triggered movements leading to reward and in conditions with no contingency between movement and reward. Our objective was to determine whether the information contained in the discharge of individual STN neurons could be related to reward and by the behavioral context in which rewards were experienced.

## Materials and methods

### Animals

Two adult macaque monkeys (G and P, *Macaca fascicularis)*, weighing 5–6 kg, were used as subjects. Experimental setup, surgical procedures and recording procedures were the same as described previously (Deffains et al., 2010). Both animals were fully trained to perform visually triggered arm-reaching movements for liquid rewards before being surgically prepared for neuronal recordings. During the training and recording periods, the monkeys were deprived of water in their home cage and received apple juice during the experiments. Unlimited water access was allowed for at least one day each week. All experiments were in accordance with the guidelines of the National Institutes of Health Guide for the Care and Use of Laboratory Animals and the French laws on animal experimentation.

### Behavioral testing conditions

**Figure 1:**
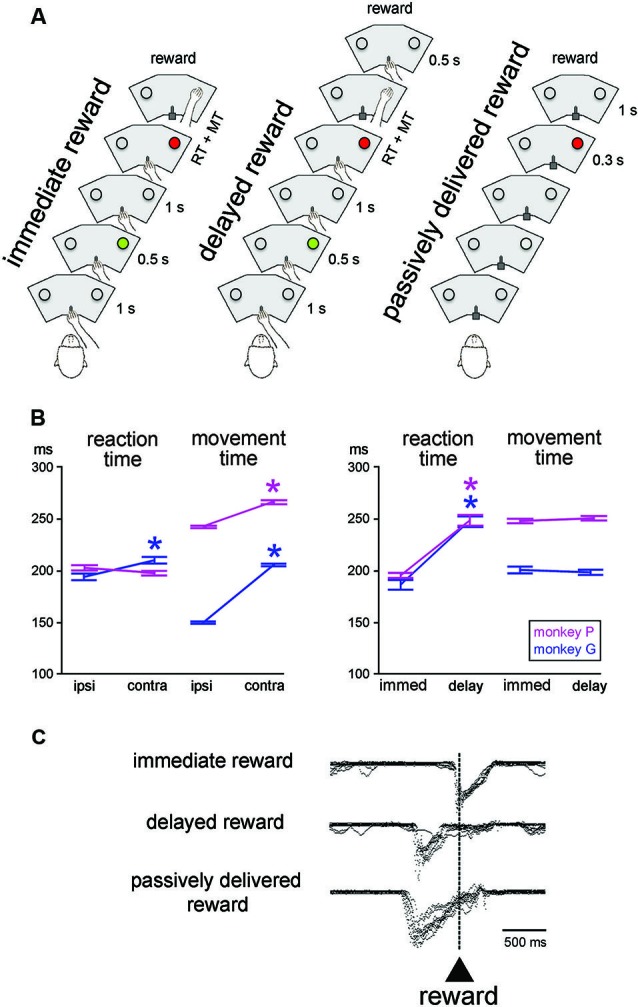
**Sequences of events and behavior in the testing conditions. (A)** In the reaching task, monkey started a trial by keeping the hand on a bar. A first visual stimulus (green light) was presented for 0.5 s at one of the two locations. A second visual stimulus (red light) was presented 1 s later at the same location. The animal was then required to release the bar and reach for the target located below the light, which it had to touch to receive a liquid reward. In the *immediate reward* condition, the reward was given immediately after correct target contact. In the *delayed reward* condition, the reward was delivered at the end of a 0.5-s interval after target contact. In the *passively delivered reward* condition, the monkey remained motionless without any access to the bar. This is a Pavlovian protocol in which a visual stimulus signaled the delivery of reward 1 s later, independently of a motor action. The three conditions were tested in separate blocks of trials. RT, MT. **(B)** Reaching task performance for the two monkeys. Each value of RT and MT was obtained by calculating the mean for all correct trials (± SEM) for the different locations of the target stimulus (*ipsi* and *contra* refer to the location of the stimulus ipsilateral and controlateral to the moving arm, respectively) and timing of reward delivery (*immed* and *delay* refer to immediate and delayed delivery of reward after target contact, respectively). An asterisk indicates that the value was significantly different between the two target locations or reward timing conditions (paired *t*-test, *P* < 0.05). **(C)** Licking behavior in the three conditions. Superimposed traces of mouth movement records are aligned on reward valve activation occurring with target contact in the immediate reward condition, 0.5 s after target contact in the delayed reward condition, and 1 s after the onset of the visual stimulus in the Pavlovian protocol, i.e., when reward was passively delivered.

The monkeys were seated in a specially designed restraining box, facing a panel 30 cm from its head. The panel contained two metal knobs (10 × 10 mm) separated by 20 cm horizontally, and two light-emitting diodes (two-colored LEDs red/green), one above each knob, at eye level of the animal (Figure 1A). An unmovable metal bar mounted at the center of the panel served as a starting point for the reaching movement. Each trial began by keeping the hand on the bar. There was no fixation point signaling the trial initiation or constraining the monkey’s eye movements. After a period of at least 1 s, a visual cue (green light) was presented randomly, either to the right or left, for 0.5 s. Cue presentation was followed by a 1-s delay period at the end of which the trigger stimulus (red light) appeared in the same location, indicating that the monkey should release the bar and touch the corresponding target. The delivery of reward (0.3 ml of apple juice) occurred immediately after target contact, under the control of a solenoid valve placed outside the experimental room. After target contact, the monkey moved back to the bar and waited for the total duration of the current trial (5 s) to elapse before a new trial began. An error trial was recorded when monkeys took longer than 1 s to initiate or execute the movement (omission trials). The two monkeys received training until they achieved a consistent correct performance rate of > 90% in the reaching task before the neuronal recording started.

In the basic task condition, reward was delivered immediately after the correct hand reaching for a target (“immediate reward”) (Figure 1A, left). We also employed another condition in which a constant delay of 0.5 s was introduced between target contact and reward delivery (“delayed reward”) (Figure 1A, middle), trials being similar to the immediate condition in all other aspects of appearance and timing. In particular, the trigger light remained illuminated until target contact, regardless of the moment of reward delivery. In a few cases, the 0.5-s delay was replaced by a 1.0-s delay when single neuron isolation was maintained long enough to complete an additional test. Because this happened infrequently, it was not possible for the monkey to predict the further lengthening of the target-reward interval. In all conditions, trials lasted 5 s so that a temporally less well-defined period began with reward delivery and ended with the cue of the subsequent trial (i.e., intervals between reward and the next cue varied from ∼2 s to 3 s in the 1.0-s delay and immediate reward conditions, respectively). The immediate and delayed reward conditions were conducted in separate blocks of 40–60 trials, the change in condition not being indicated by any explicit cues. However, because of the block design, it did not take monkeys many trials to adjust their expectation after a switch in testing condition. Before the recording experiments started, both monkeys were well-trained in the immediate reward condition, whereas the delayed reward condition was used only occasionally during recording sessions.

We also employed a condition in which the liquid reward was delivered in a passive manner after a period of 1 s after one LED was illuminated with a red light (Figure 1A, right). This was a Pavlovian conditioning procedure in which reward was not contingent on behavior (Apicella et al., 1997). In this testing condition, the sliding door located at the front of the restraining box was closed to prevent manual access to the panel. In addition to the testing of the monkeys in the Pavlovian protocol, we also delivered the liquid at unpredictable times, in the absence of any cue to precisely time when the reward would be delivered. The change from instrumentally to passively delivered reward was indicated by the experimenter entering the experimental room to open or close the sliding door of the box. Conversely, there was no signal indicating that the Pavlovian protocol was about to be changed to a block of trials in which reward was delivered alone. Passively delivered reward conditions were presented using the same trial duration as used in the reaching task, thus preserving the overall temporal structure of the testing condition.

### Neuronal recordings

On completion of training, each monkey underwent sterile surgery under sodium pentobarbital anesthesia. An opening was made in the skull over the left hemisphere and a stainless steel recording chamber (25 mm OD) was implanted over the hole, its center being aimed at the anterior commissure (AC), approximately 5 mm anterior to the rostral pole of the STN. The chamber was held in place by dental acrylic anchored with stainless steel screws drilled into the skull. Two stainless steel cylinders were also embedded in dental acrylic for subsequent head fixation during recording sessions. Antibiotics and analgesics were administered after surgery. Extracellular activity of single neurons was recorded with tungsten microelectrodes, as described previously (Deffains et al., 2010). In both monkeys, with their heads restrained while performing the behavioral tasks, neuronal recordings were carried out first from striatum. After several months of recording mainly targeted at the putamen, finding the accurate location of the STN was relatively easy using the striatal tracks, particularly at the level of the posterior putamen. Parallel electrode tracks were then made vertically, through the thalamus, zona incerta, STN and substantia nigra pars reticulata, in that order, the transition between these structures being obvious because of grossly different spontaneous neuronal activity. The electrode was driven by a hydraulic microdrive (MO-95; Narishige, Tokyo, Japan) through a stainless steel guide tube, which was used to penetrate the dura. After penetration of the dura, the electrode was advanced until the dorsal border of the STN was identified by an increase of background noise and typical large-amplitude irregular spike activity after passing the white matter area below the thalamus (i. e., fields of Forel) and zona incerta (Matsumura et al., 1992; Wichmann et al., 1994; Isoda and Hikosaka, 2008). Signals from neuronal activity were conventionally amplified, filtered (bandpass, 0.3–1.5 kHz), and converted to digital pulses through a window discriminator. During the recording of any neuron, activity in the immediate reward version of the reaching task was generally studied first, and, if the isolation could be sustained for a sufficient period of time, the tests were continued in the other conditions. It needs to be pointed out that the relative lack of stability of recording in the primate STN did not always ensure the isolation of individual neurons over successive trial blocks for different conditions (Wichmann et al., 1994). We have succeeded in helding stable neurons for two or more blocks in only a few cases. Presentation of visual stimuli, delivery of reward, collection of movement parameters, mouth movements and single-neuron activity were controlled by a computer, using custom software written by E. Legallet.

### Data analysis

Performance in the reaching task was assessed by measuring the reaction time (RT), i.e., the time between the onset of the trigger stimulus and release of the bar, and the movement time (MT), i.e., the time taken to contact a target after releasing the bar. The analysis included data from all correctly performed trials and all recording sessions, excluding error trials (omissions of the trigger stimulus) and premature responses (RTs < 100 ms). The tube conducting reward liquid to the spout positioned directly in front of the monkey’s mouth was equipped with a strain gauge circuit with which we monitored the licking movements as analog signals (sampling rate: 100 Hz). The timing characteristics of the mouth movements that monkeys performed in the different conditions were assessed off-line by single-trial analysis.

To analyze neuronal correlates of the initiation of movement, we calculated the mean firing rate in a 300-ms time window extending from 200 ms before movement onset until 100 ms after movement onset, called the “perimovement period”, and in another 300-ms time window extending from 400 to 700 ms after target contact, called the “postreward period”. The mean discharge rate in each task period was compared with that in the control period (the 1 s duration before the cue onset) to examine whether the neuron showed significant task-related activities. If the mean discharge rate in a given period was significantly different from that in the control period (two-tailed Student *t*-test, *P* < 0.05), the neuron was considered to show task-related activity in that period. We tested for changes in activity during these two period for each recorded neuron.

The selectivity of the task-related activity for a particular location of the target stimulus was judged to be present if the magnitudes of the perimovement activity were significantly different between the two locations (two-way ANOVA, period (control, perimovement) × location (left, right), *p* < 0.05).

To determine the response latency of a neuron to a particular task event, onset and offset times of statistically significant changes in activity were assessed using a previously established procedure based on a sliding time window analysis (Deffains et al., 2010). Briefly, baseline activity was determined in the 1-s period that preceded the onset of the cue (control period). A test window of 100 ms was moved in steps of 10 ms, starting at the onset of the cue. We then compared activity from the baseline period to activity in the sliding window. Neurons showing a statistically significant difference in activity during ≥ 20 consecutive steps (Wilcoxon signed-rank test, *P* < 0.05) were considered as modulated. The latency of a significant change in neuronal activity was defined as the beginning of the first of 20 consecutive steps showing a significant difference as against the baseline activity during the control period.

In addition to the assessment of activity changes of the individual STN neurons, we also summed activity of all neurons tested in a given condition or for a particular location of the target stimulus and made population histograms. For each neuron, a normalized perievent time histogram was obtained by dividing the content of each bin by the number of trials. The population histogram was obtained by averaging all normalized histograms referenced to a particular event. These histograms were constructed from neurons recorded in monkeys P and G in each testing condition.

### Histology

We confirmed our recording sites by histological verification in one monkey, whereas the location of recorded neurons was determined solely on the basis of the electrophysiological information concerning the boundaries of the STN and surrounding structures in the other monkey. After the experiments had been completed, monkey P was sacrificed with an intravenous overdose of pentobarbital and perfused transcardially with 0.9% saline followed by a fixative (4% paraformaldehyde, pH 7.4 phosphate buffer). The brain was cut in 50-μm coronal sections, mounted on slides, and stained with cresyl violet. We identified the recording sites that had been marked with small electrolytic lesions in and around the STN at the end of neuronal data collection. Electrode penetrations were then reconstructed in serial sections through the STN by referring to the marking lesions.

## Results

### Task performance data

The mean RT and MT for different target locations and reward timing for each monkey are presented in Figure 1B. Consistent with previous reports from our lab (Ravel et al., 2006; Deffains et al., 2010), RTs to target stimuli presented contralaterally to the moving arm were slightly but significantly longer than RTs to ipsilateral target stimuli in monkey G (Student’s *t*-test, *P* < 0.01), whereas the effect of spatial location on RT was no significant in monkey P (*P* > 0.05). On the other hand, MTs for contralateral movements were longer than MTs for ipsilateral ones in both monkeys (*P* < 0.01). It is possible that monkey P used prior information about the target location efficiently to prepare the movement and then improve speed during initiation, thus explaining the lack of spatial response bias in the initiation phase of movement in this animal.

As also shown in Figure 1B, comparing the RTs obtained during blocks where the reward was delivered immediately after target contact with those where the reward was delayed by 0.5 s after target contact, yielded significant longer RTs in the latter condition in both monkeys (two-way ANOVA) with the factors target location and reward timing, effect of reward timing: monkey G: (*F* (1, 401) = 73.82, *P* < 0.01; monkey P: (*F* (1, 364) = 96.16, *P* < 0.01). In contrast, for MT, there was no significant difference between the two conditions (monkey G: *F* (1, 401) = 0.47, *P* > 0.05; monkey P: *F* (1, 364) = 0.65, *P* > 0.05). There was no interaction between target location and reward timing regarding RT and MT in monkey G (*P* > 0.05), whereas such interactions were detected for RT (*P* < 0.05) and MT (*P* < 0.01) in monkey P. These findings indicate that monkeys took longer to initiate reaching movements when the timing of the reward outcome was delayed. These results differ from what was found in a previous study, using a similar reaching task, in which we reported that neither RT and MT were influenced by the delayed delivery of reward after target contact (Ravel et al., 2001). This lack of concordance could be attributed to differences in training levels, the small sample of neurons tested in the delayed reward condition in the present study did not allow enough training. In a few cases (1 and 4 trial blocks in monkeys G and P, respectively), a 1-s delay was employed in order to further lengthen the period of expectation of reward after target contact. This test was only used when stable recordings were obtained on neurons that were modulated during the target-reward interval of 0.5 s. In monkey P, two-way ANOVA performed with target location and reward timing (immediate, delay 0.5-s, delay 1.0-s) as factors revealed a significant main effect of reward timing on RT (*F* (2, 266) = 31.01, *P* < 0.01), so that RT was longer in the delay 1.0-s than the delay 0.5-s conditions. In contrast, the MTs varied insignificantly by reward timing (*F* (2, 266) = 1.97, *P* > 0.05). When monkeys were tested with delayed reward conditions, whether a 0.5-s or a 1.0-s delay, they moved immediately their hand back to the bar after having touched the target and then waited for the delay before reward. In this regard, there were no noticeable differences in gross behavior between immediate and delayed reward conditions while the animal was resting the hand on the bar in order to initiate the next trial.

Representative mouth movement recordings are illustrated in Figure 1C. They were essentially the same as those described in earlier studies using similar behavioral situations (Ravel et al., 2001; Apicella et al., 2009). The monkey started to lick the spout on or slightly before target contact in the instrumental task, regardless of the timing of reward, and these movements were prolonged until the receipt of liquid when a 0.5-s delay was introduced between target contact and reward delivery. Licking movements occurred during the 1-s interval separating the presentation of the visual stimulus from the delivery of reward, indicating that the stimulus served as a trigger for mouth movements in anticipation of the upcoming reward. This served as a well-established behavioral marker of the Pavlovian stimulus-reward association.

### General

While the monkeys performed the reaching task, we recorded from 74 neurons in the STN (51 and 23 from monkey G and P, respectively) with a background firing rate of 23.5 ± 16.4 spikes/s (mean ± SEM). Most neurons (*n* = 59) had medium to high firing rates (mean 28.6, range 10.3–71.3), a small number (*n* = 15) had firing below 10 spikes/s (mean 3.4). The firing characteristics of STN neurons were similar to what has been reported previously in behaving monkeys in terms of spiking irregularity and baseline firing rate (Matsumura et al., 1992; Wichmann et al., 1994; Isoda and Hikosaka, 2008).

**Figure 2:**
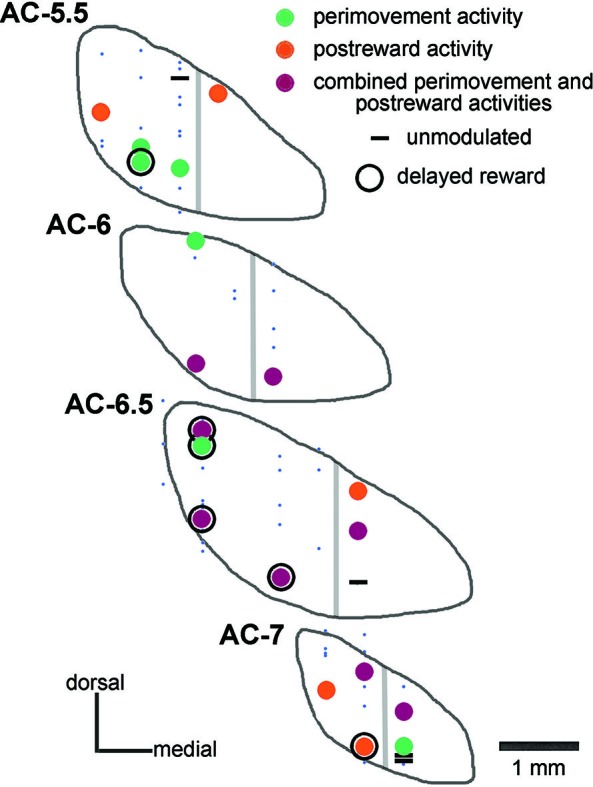
**Histological verification of recording sites in the STN of one monkey.** Data from monkey P are plotted on coronal sections of the STN referenced to the AC. Neurons studied during the performance of the reaching task were classified as being modulated around the initiation of movement (*perimovement activity*), after the delivery of reward (*postreward activity*), at both periods of the task (*combined* *perimovement and postreward activities*), or without any change in their firing in these two periods (*unmodulated*). Neurons whose recordings were not sufficiently stable to permit adequate testing are represented by small blue dots. Neurons displaying a sustained change in activity when a delay between target contact and reward delivery was introduced are surrounded by a black circle (*delayed reward*). Gray lines indicate the approximate boundary of the “sensorimotor” and “cognitive-limbic” parts of the STN.

Based on the location of recording sites identified histologically in monkey P, these neurons were recorded between 5 and 7 mm posterior to the AC, over the mediolateral extent of the STN, except its most medial part. A summary plot of all neurons recorded is shown in Figure 2, including those that could not be recorded for enough number of trials. On the basis of histological analysis of electrode tracks in this monkey, it appears that we failed to adequately sample the most medial portion of the nucleus in its more anterior parts. As regards the recording sites of well isolated neurons, the two major groups of task-related changes in activity (i.e., those occurring during the perimovement period and/or the postreward period) did not show clear regional differences in the STN explored.

In general, STN neurons exhibited a variety of changes in activity during various periods of the reaching task, with some neurons being modulated before the onset of the movement and others being modulated later, after the monkey’s hand contacted the target and was immediately followed by the delivery of reward. These modulations could consist in either excitation or inhibition. As we point out below, the same neurons often displayed changes in activity during two distinct task periods, namely around the initiation of movement and after target contact.

### Activity after target contact

**Figure 3:**
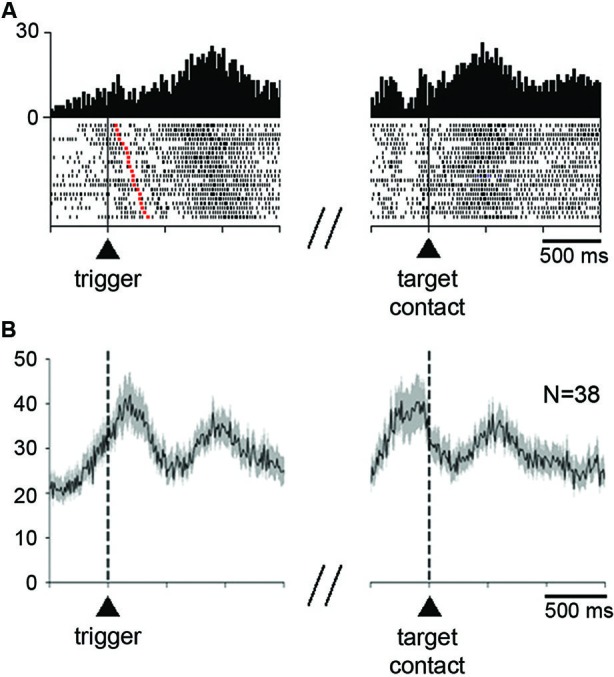
**Neuronal activity in the STN during the performance of the reaching task. (A)** An example of neuron showing an increase in firing rate before the initiation of movement and after target contact immediately followed by reward delivery. Each dot in the raster displays represents one neuronal impulse, and each line of dots represents the neuronal activity during a single trial. Dot displays and perievent time histograms are aligned on the onset of the movement triggering stimulus (left) or the contact with the target (right), which are marked by vertical lines. The raster was sorted in order of increasing RT, i.e., increasing interval from trigger onset to the onset of movement (red squares in each row). Histogram scale: impulses/bin. Binwidth for histograms: 20 ms. **(B)** Modulation of population activity in the reaching task. Only neurons showing increases in firing rate after target contact, pooled from both monkeys, are included in this analysis. Average activity is aligned on the trigger stimulus (left) or target contact (right), which are marked by vertical dashed lines. Vertical scale: impulses/s. N: number of neurons included for the population histogram.

The activity of 70% (52/74) of neurons (13 of 23 in monkey P, 39 of 51 in monkey G) was significantly modulated after reaching toward the correct target. Figure 3A shows an example of neuron with increased activity after target contact (right part). This neuron also showed increased activity, although to a weaker degree, before the initiation of movement (left part). As is demonstrated by this case, the temporal linkage to movement onset was clearly apparent by aligning neuronal activity on bar release. The activity was rapidly decreased immediately after the onset of movement and increased again after movement termination, lasting more than 1 s after target contact. About three quarters of the detected changes occurring after target contact consisted of increases and one quarter of decreases.

As illustrated in Figure 3A, the majority of neurons (35/52) significantly modulated after target contact also showed a significant change in activity around the initiation of movement. Thirty three neurons changed their activity in the same direction for both movement initiation and after target contact (28 increases, 5 decreases), whereas only 2 neurons had opposite changes in activity (an increase for movement initiation and a decrease after target contact). Changes in activity occurring specifically after target contact were observed in 17 neurons (10 increases, 7 decreases) and 15 neurons showed an exclusive change in activity around the initiation of movement (10 increases, 5 decreases).

We examined the time course of activity changes at the level of the whole sample of neurons modulated during the postreward period (Figure 3B). Only neurons showing increases in their firing rate were included in this population average. The average activity reached a first peak of increased activity slightly after the presentation of the trigger stimulus that elicited movements. A second peak of increased activity occurred later after target contact. It is noticeable that this latter peak was well located in the 300-ms time window we have chosen for analysis (i.e., 400–700 ms after target contact), indicating that the majority of activations following target contact were relatively late increases.

The relative late onset of peak firing after target contact suggests that changes in STN neuronal activity did not occur directly in response to target contact. Also, peak firing did not appear to be associated with the monkey’s movement back to the resting bar. Although a possible relationship to the consumption of liquid must be taken into account, it seems that the motor aspects of orofacial activity were not the only explanation, particularly for those neurons which began to be modulated with a relatively long latency after target contact. This explanation, however, does not hold for other neurons that became active earlier, during the reaching movement, a task period in which preparation and initiation of mouth movements took place.

**Figure 4:**
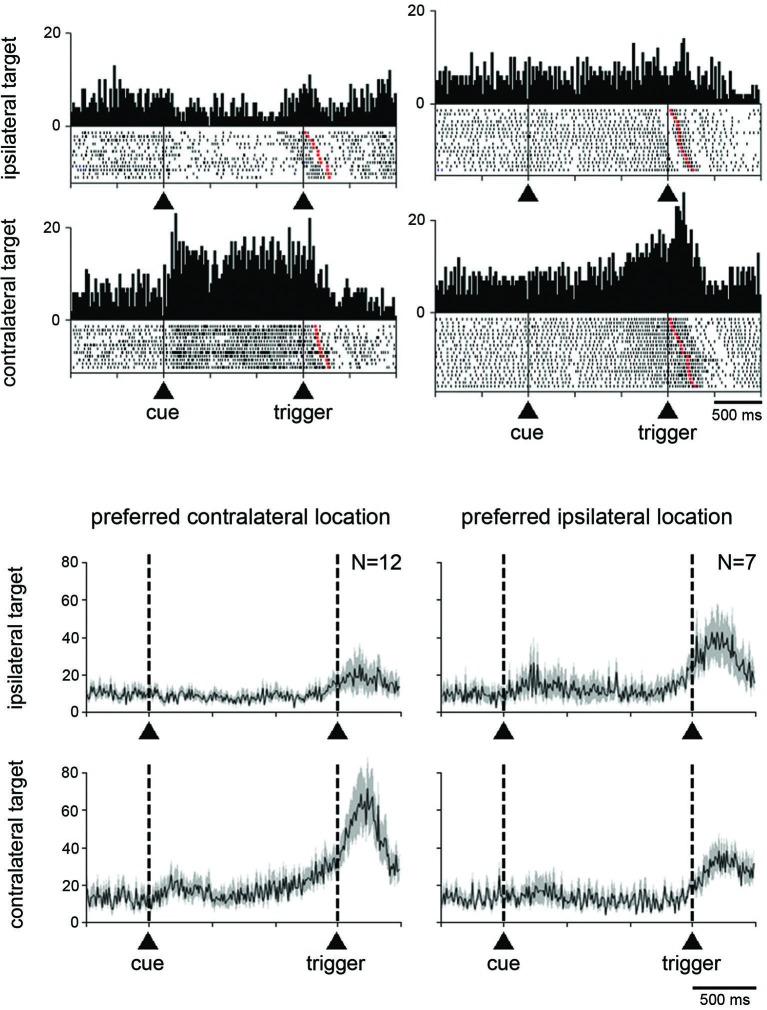
**Neuronal activity in the STN related to the spatial location of the target stimulus. (A)** Two examples neurons showing a spatial preference for target stimuli presented on the side contralateral to the moving arm. Data were separated off-line according to the location of the target stimulus. Same conventions as in Figure 3A. **(B)** Modulation of population activity dependent on the location of the target stimulus. Populations included all neurons with a spatial selectivity in the magnitude of their modulation, separately for each preferred location. The data were taken from monkey G. Same conventions as in Figure 3B.

We next examined whether the magnitude of the change in activity during the perimovement period was affected by the location of the target stimulus and/or the direction of the associated movement. Target location had a significant effect (*P* < 0.05; two-way ANOVA) on perimovement activity in 22 of the 74 neurons tested (19 and 3 neurons in monkeys G and P, respectively). Among them, 19 showed increases in activity around the initiation of movement and 3 showed decreases. The fact that most of the neurons sensitive to the location of the target stimulus were recorded in monkey G in which a spatial response bias was observed behaviorally (Figure 1B), suggests that a contralateral preference in STN modulations could emerge in parallel with slow movement initiation in the contralateral direction. We found that 14 of the 22 neurons displaying spatially selective modulations preferred the contralateral stimulus location, and 8 the ipsilateral one (*P* < 0.05; two-way ANOVA followed by Tukey’s test). This is illustrated by data from two example neurons classified as spatially selective in Figure 4A. The first neuron (left part) is an example of directional preparatory activity, the selective activation being manifested by a sustained increase in discharge rate during the delay period prior to contralateral movement, whereas a corresponding reduction in activity occurred with ipsilateral movements. The other neuron (right part) showed a contralateral-selective activation in advance of trigger presentation and until movement onset. It is noteworthy, in the two example neurons, that the change in activity terminated with the onset of movement, rather than the presentation of the stimulus triggering movement. Moreover, a phasic component occurring just before the movement onset was still visible even in the case of ipsilateral movements, suggesting the presence of a neuronal signal related to movement initiation that did not depend on the spatial features of the task.

Separate population histograms were constructed from all neurons showing target location selectivity (Figure 4B). This analysis was confined to data from neurons recorded in monkey G showing increased activity during the perimovement period and in which a significant effect of spatial location was detected. Despite the small number of neurons, the spatial preference in terms of magnitude of change in the average activity was obvious when the stimulus was presented contralaterally to the reaching arm. As shown in Figure 4B, the selectivity for ipsilateral target location appeared less evident at the level of the population average, compared to contralateral location.

### Influence of delaying the reward after target contact

**Figure 5:**
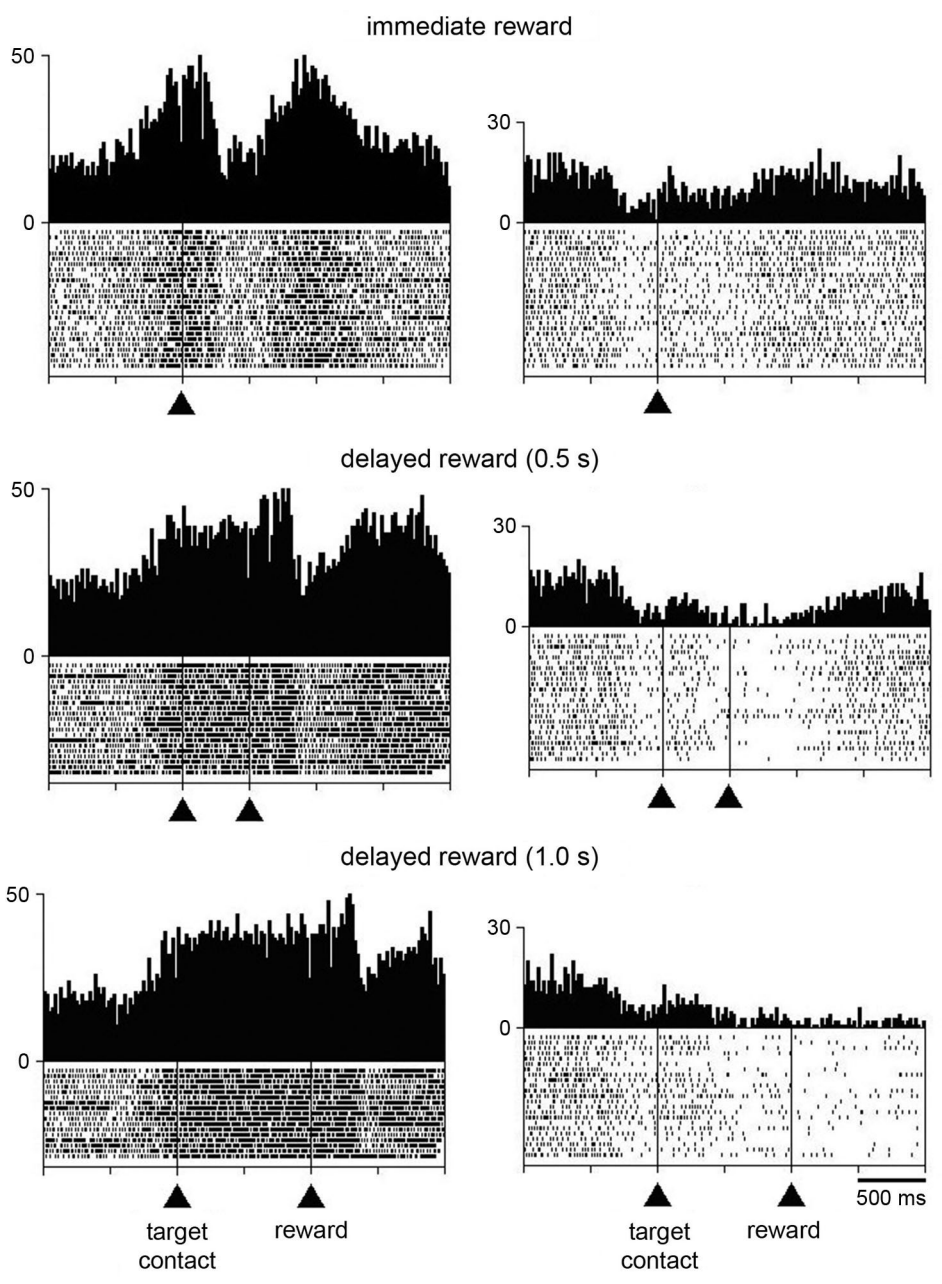
**Influence of delaying the time of reward delivery in the reaching task in two STN neurons.** For each recorded neuron, the change of condition occurred over three successive blocks of trials in which reward was delivered immediately, 0.5 s, or 1 s after target contact. Same conventions as in Figure 3A.

Additional tests were performed for characterizing changes in activity following target contact. In particular, we wanted to separate in time movement termination from the receipt of liquid by delaying the reward for 0.5 s, occasionally 1 s, after the contact of the monkey’s hand with the target. In 21 neurons (8 and 13 from monkeys P and G, respectively), single neuron isolation could be successfully maintained during recording in both the immediate and delayed reward conditions. Of these, 16 neurons showed a sustained change in activity through the delay between target contact and reward delivery, consisting of increases and decreases in activity in 10 and 6 neurons, respectively. For 5 of the 16 neurons showing a sustained change in their activity during the 0.5-s delay (2 increases, 3 decreases), data were also collected when the target–reward interval was further lengthened to 1 s and we verified that the change in activity was prolonged accordingly. This is consistent with previous studies demonstrating that STN neurons recorded in behaving monkeys can display sustained changes in activity as a possible reflection of a state of expectation of the delivery of reward (Matsumura et al., 1992; Darbaky et al., 2005). Figure 5 shows data from two neurons which have been fully tested in three successive trial blocks, illustrating activity profiles dependent on the duration of the delay between reaching the target and receiving the reward. The rasters in the left panels represent the activity of a neuron which displayed elevated activity during the delay. The same pattern of activity is seen in the neuron illustrated in the right panels, but consisting in a suppression of activity during the delay. In both cases, modulations occurring during the delay began just before target contact and continued until reward delivery. Despite the limited size of our data set, it is noticeable that sustained changes in activity before reward delivery were frequently found in the ventral half of the nucleus (Figure 2) which has been shown previously to contain neurons related to reward expectation in monkey (Matsumura et al., 1992).

In order to investigate whether the task-related changes in STN neuronal activity showed a dependency on reward timing, we tested the activity of each neuron in the perimovement period with a three-way ANOVA with factors: period × target location × reward timing. Of the 21 neurons recorded in both the immediate and delayed reward conditions, 16 were modulated in a similar manner and 5 showed a significant difference in the magnitude of perimovement activity between the two conditions. Specifically, 3 neurons were modulated more strongly when the reward was delivered immediately after the target contact, whereas 2 neurons were modulated more strongly when monkeys received reward 0.5 s after the target contact. It therefore appears that there was no systematic relationship between the level of modulation and reward timing, suggesting that changes in movement speed were not linked to perimovement changes in STN firing as a function of reward timing. On the other hand, a significant difference in the magnitude of postreward activity between the two conditions was detected in 15 of the 21 neurons, with 10 of them being modulated more strongly when the reward was delivered immediately after the target contact and 5 neurons being modulated more strongly when reward was delayed by 0.5 s. This suggests that modulations of postreward activity can be explained, at least for some STN neurons, by the subjective value of reward which decayed as the time to its delivery was delayed. The small number of neurons tested with the 0.5-s and 1.0-s delays prevented a similar analysis.

### Influence of delivering rewards not contingent upon instrumental reactions

**Figure 6:**
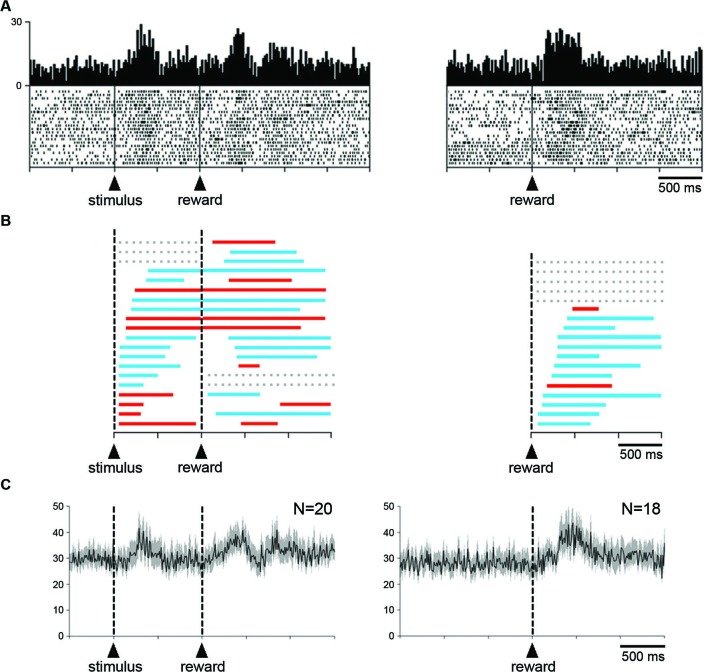
**Influence of reward delivery that is not contingent on behavior on the STN neuronal activity. (A)** An example of neuron showing an increased in firing rate after the visual stimulus that signals the delivery of reward and after the reward itself under the Pavlovian condition (left). The same neuron also displayed an increased in firing rate after the delivery of reward not signaled by a predictive cue (right). Same conventions as in Figure 3A. **(B)** Time course of changes in activity of all neurons tested in the Pavlovian protocol (left) and during the delivery of reward alone (right). Each colored horizontal line represents the period with a statistically significant increase (blue) or decrease (red) in activity for a single neuron. Lines are ordered according to onsets of activity change after the presentation of the visual stimulus or the delivery of reward. Gray horizontal dashed lines indicate a lack of significant change in discharge rate after a given event. **(C)** Modulation of population activity. Population included all neurons (i.e., both increases and decreases) recorded in the two testing situations. Same conventions as in Figure 3A.

The relationship of changes in neuronal activity to the expectation and detection of reward was further examined when the liquid was delivered outside of the reaching task. Twenty neurons (16 and 4 neurons in monkeys G and P, respectively) were studied in the Pavlovian protocol. Figure 6A (left panel) shows a neuron that displayed two consecutive components of neuronal modulation, i.e., a brief increase in firing after the presentation of the visual stimulus and another increase after the subsequent delivery of reward. The same neuron was also recorded when the reward was delivered alone, in the absence of the preceding stimulus (right panel), and an increase in firing was still detected after this single event.

A notable feature of STN neurons recorded in the Pavlovian protocol is the substantial variability in the temporal profile of their changes in activity. To quantitatively assess this, the times of onset and offset of modulation were determined for each neuron with the use of the sliding window procedure (see Section Materials and Methods). The distribution of temporal profiles of activity relative to stimulus onset for every recorded neuron is presented in Figure 6B (left). We found that 17 neurons were significantly modulated after the presentation of the visual stimulus (10 increases, 7 decreases), the remaining 3 neurons being modulated only after the delivery of reward (2 increases, 1 decrease). As can be seen in this figure, the change in activity after stimulus onset was maintained in the time period immediately following stimulus onset for 16 neurons and even extended beyond reward delivery for 15 neurons. Two neurons were modulated only after the presentation of the stimulus (2 increases). Some activity changes began after stimulus onset, lasted before reward delivery and then restarted later after the delivery of reward, whereas others persisted through the delay until the delivery of reward. Overall, it appears that the distributions of onset of modulation overlapped substantially among neurons with task-related increases and decreases in activity. In 10 neurons, the receipt of reward produced a change in activity of the same sign as the change following stimulus onset, whereas 5 neurons had bidirectional changes in activity. These observations are consistent with the idea that STN neurons were not exclusively sensitive to the reinforcement of an instrumental response. We examined the population activity for the sample of 20 neurons recorded in the Pavlovian protocol (Figure 6C). Considering the complex pattern of changes in activity described above, we combined all neurons, regardless of their increase and/or decrease in firing rate. Two phases of increased activity were visible after each task event in the population average. The first phase appeared relatively homogeneous, whereas the second phase included multiple components.

Finally, it was of special interest to examine STN neuronal activity when the monkey received reward without any predictive cues. Among a sample of 18 neurons (3 and 15 neurons from monkey P and G, respectively), 13 displayed changes in activity after the delivery of reward (11 increases, 2 decreases). The remaining 5 neurons showed no detectable change in activity during the test. As mentioned above, the example neuron shown in Figure 6A (right) increased its activity in response to reward given alone. The temporal parameters of the changes in activity were also analyzed for each neuron tested in this condition and the results of this analysis are illustrated in Figure 6B (right). It can be seen that the increases in firing occurred more frequently than the decreases. We then performed a population analysis for these 18 neurons and by comparing it with the average activity obtained in the Pavlovian protocol, it appears that the modulation in response to the delivery of reward was more homogeneous in the former condition.

## Discussion

Although clinical and neurophysiological evidence has long pointed to the role of STN in regulating motor function, recent studies have also implicated this nucleus in the processing of reward-related information. Consistent with this idea, we found that STN neurons displayed changes in activity after the delivery of reward, regardless of the need to make a movement to obtain reward. When monkeys were actively engaged in target reaching, the reward signals carried by STN neurons were often combined with modulations around the time of movement onset, demonstrating that STN neurons are sensitive to the movement and its reward outcome. These observations confirmed earlier findings from only a single monkey performing a reaching task to obtain reward (Darbaky et al., 2005). In the present study, we have provided further details of reward-related changes in STN activity under varying contextual conditions, i.e., during instrumental and Pavlovian conditioning tasks and even in the absence of any reward predictive cue. In addition, our findings have emphasized the presence of STN neurons exhibiting sustained changes in activity in advance of reward whose delivery was delayed in the reaching task, indicating that STN neuronal activity is influenced by the state of expectation of future rewards. Altogether, these results obtained in non-human primates are consistent with the idea that the STN is a component of basal ganglia circuitry that mediates motivational processes.

### Reward-related activity in the STN

Even if increasingly more clinical and animal lesion studies are highlighting the role of the STN in reward circuitry, only a few studies of neuronal activity in the STN have documented the sensitivity of individual neurons to the delivery of reward during the performance of motor tasks in both rodents (Teagarden and Rebec, 2007; Lardeux et al., 2009, 2013) and monkeys (Matsumura et al., 1992; Darbaky et al., 2005). Previously, we have shown in a preliminary study that STN neuronal activity was modulated by reward delivered after an instrumental response, and preliminary work done at that time indicated that reward-related modulations were still detected when reward was passively delivered in a Pavlovian manner (Darbaky et al., 2005). The activity changes reported here confirm these preliminary findings and further indicate that STN neurons were responsive to unpredicted deliveries of reward. It therefore appears that STN neurons have firing rates that are sensitive to appetitive events themselves, whether or not a motor response was required to obtain reward and even outside of Pavlovian or instrumental response control. However, as we discuss below, circumstances in which rewards are passively experienced by animals do not exclude a possible influence of motor constraints to consume the liquid.

Earlier reported changes in STN neuronal activity after reward delivered have been reported in rats performing instrumental tasks (Teagarden and Rebec, 2007; Lardeux et al., 2009, 2013) and are comparable to those reported here in monkeys. In addition, Lardeux et al. (2009) have further demonstrated that STN activity may be related to reward quality (i.e., different sweetness of a liquid sucrose reward). These same authors have recently reported that different populations of STN neurons were modulated by the delivery of an appetitive liquid or administration of a psychoactive drug (Lardeux et al., 2013), suggesting that distinct neuronal circuits within the STN mediate the positive value of stimuli.

Several basal ganglia structures with neuronal activity linked to the rewarding significance of conditioned stimuli as well as to reward itself have been extensively investigated in both rodents and monkeys (Schultz et al., 2000; Hikosaka et al., 2006). It appears that the STN also contributes to the processing of motivational information. How reward sensitivity is encoded in the STN compared with that found in other components of the basal ganglia circuitry, particularly the striatum, remains to be clarified.

Although our results showed, at the single-neuron level, the contribution of the STN in the detection of rewarding events, they did not allow to firmly establish whether the observed changes in activity were related to the hedonic nature of the reward. In particular, the possibility that the reward-related activity encoded some aspects of orofacial behavior was not totally eliminated. In an attempt to dissociate mouth movements from the delivery of reward *per se*, we have examined the timing characteristics of licks at the spout during neuronal recordings. As already pointed out in our previous study (Darbaky et al., 2005), the reward-related activity was not directly related to the licking patterns in terms of onset and duration. For example, in the reaching task, the majority of modulations of STN neuron firing which occurred after target contact were relatively late changes, suggesting that they were unlikely to be coupled to preparation or initiation of mouth movements which began earlier. We cannot exclude, however, that they could be involved in later phases of liquid consumption, such as swallowing. Moreover, the variability in the time course of the observed changes in neuronal activity during Pavlovian conditioned behavior challenges the notion that STN neurons may encode the highly stereotyped pattern of licking movements elicited in this condition.

### Heterogeneity of reward-related activities in the STN

Consistent with previous electrophysiological studies, we have found that STN neurons were either excited or inhibited by rewarding events, increases being more common than decreases. Interestingly, the prevalence of increases in STN neuronal activity became particularly evident when switching from a Pavlovian association between stimulus and reward to a situation in which reward was not signaled by a cue, thus suggesting a change in the way that the reward itself is processed by STN neurons. For comparison with our earlier study (Darbaky et al., 2005), it was also noticed that increases in STN neuronal activity were more frequent when reward was given outside of a learning context. In the rat experiments, modulations of STN firing following reward delivery also consisted of either an increase or a decrease in firing (Teagarden and Rebec, 2007; Lardeux et al., 2009, 2013), although the respective proportions of changes in opposite direction could be different from one study to another, and an influence of context on reward-related activities was also highlighted in the case of changes in reward values (Lardeux et al., 2009, 2013). Because behavior-related increases and decreases in the activity of STN neurons are assumed to have opposing effects on behavioral output, it is important to understand the significance of these opposing changes in activity. Indeed, increases in STN activity are thought to suppress movement execution by increasing inhibition of thalamic and brainstem targets via basal ganglia output structures and the reverse may occur in the case of decreases in STN activity.

### Changes in STN neuronal activity related to specific aspects of target reaching

Our findings have shown two main and temporally distinct patterns of task-related activity in the STN during performance in the reaching task, one occurring around the initiation of movement and the other after target contact, immediately followed by reward delivery. Previous studies in behaving rats have also shown that most neurons in the STN carry signals related to both motor behavior and reward outcomes (Teagarden and Rebec, 2007; Lardeux et al., 2009, 2013). These observations point to an influence of motor and motivational aspects of task performance at the single-neuron level, possibly reflecting the fact that reward signals generated by STN neurons are linked to the performance of specific actions.

In the present study, we have examined the involvement of the STN in motor aspects of performance specifically regarding the visuospatial features of the reaching task. We found that a number of STN neurons displayed preferences for one location of the triggering stimulus and/or direction of its associated motor response, most of them preferring the location and/or direction opposite to the moving arm. In at least one monkey, this spatial preference was accompanied by a variation in the speed of the motor response to the stimulus. A link between STN neuronal activity and spatial information provided by stimuli eliciting movements in various directions has been reported in previous monkey studies (Georgopoulos et al., 1983; DeLong et al., 1985; Isoda and Hikosaka, 2008). However, it is unknown whether this spatial selectivity was related to the location of the trigger stimulus and/or the direction of the movement associated with that stimulus. Indeed, in these studies (including our own), the motor response was directed toward the spatial location of the trigger stimulus, so that it cannot be established whether the observed changes in activity were dependent on the “sensory” or “motor” constraints. Only a few studies have attempted to dissociate these two aspects while studying task-related neuronal activity in basal ganglia (Alexander and Crutcher, 1990; Ravel et al., 2006). However, in rats trained to make a constant motor reaction (i.e., cessation of lever pressing) to different spatial locations of a movement-triggering stimulus, no influence of location was reported on the activity of STN neurons (Lardeux et al., 2009).

### Changes in STN neuronal activity related to reward expectation during task performance

We have found that STN neurons may exhibit persistent changes in firing throughout a time interval introduced between correct target contact and reward delivery in the reaching task. These findings are consistent with previous observations in monkeys that showed the influence of variations in reward timing on STN activity (Matsumura et al., 1992; Darbaky et al., 2005). In addition, we showed that monkeys were sensitive to the presence of a reward delay after target reaching, suggesting that they discounted the value of a reward when it is delayed in time. We found that few neurons exhibited non-systematic differences in firing around the initiation of movement which leads to an immediate or delayed reward. On the other hand, a relatively large number of neurons showed stronger changes in activity following reward delivered immediately after completion of movement, compared with delayed reward, suggesting that they may participate in the time-discounted encoding of reward value. In the present study, sustained changes in activity were extended when the target-reward interval was prolonged to 0.5 to 1 s, suggesting that this activity may be interpreted as reflecting a representation of outcomes which is crucial for the control of reward-guided behavior. Again, we cannot exclude completely the alternative explanation that the observed changes in neuronal activity may reflect preparation to consume the liquid (Roesch and Olson, 2003). The observed modulations in STN neuronal activity did not appear to be related to the simultaneously occurring mouth movements, suggesting that these modulations might be related more to reward expectation than to motor preparation, but this needs to be further clarified. In addition, our task manipulation did not allow a clear-cut dissociation between motivation and attention (Maunsell, 2004). Since STN has been shown to be involved in attention (Baunez and Robbins, 1997, 1999), we cannot rule out a contribution of attentional processes in the neuronal changes reported here. Additional work is needed to clarify this issue.

### Is reward-related activity confined to specific regions of the STN?

Based on anatomical studies of corticosubthalamic circuitry, it is thought that regions of the STN can be functionally delineated along distinct territories. Although neuroimaging data do not provide the spatial resolution required to examine these distinctions, recording studies at a single-neuron resolution level may be helpful to analyze the fine distribution of neurons displaying specific response properties. Previous studies have reported that neurons related to limb movements are located primarily in the dorsolateral STN region (Georgopoulos et al., 1983; DeLong et al., 1985; Wichmann et al., 1994), whereas neurons related to visuomotor functions were generally located more ventrally (Matsumura et al., 1992; Isoda and Hikosaka, 2008). Although anatomical connectivity defines an organization in subterritories (Groenewegen and Berendse, 1990), the functional evidence is less clear for the rodent STN for which researchers refer to task-related activities without attempting to parcel out the subdivisions (Teagarden and Rebec, 2007; Lardeux et al., 2009, 2013). Despite some limitations on the extent of the area over which we recorded STN neurons, our results based on histological examination in one monkey did not show regional differences that were particularly obvious in the pattern of distribution of task-related neurons. In particular, neurons sensitive to reward were scattered throughout the parts of the STN explored, without preferential location in the ventromedial part, which receives inputs from the orbitofrontal cortex and anterior cingulate cortex and is considered the « limbic » part of the STN in primates (Takada et al., 2001; Karachi et al., 2005; Haynes and Haber, 2013). This observation confirms what had been previously noted in our preliminary study (Darbaky et al., 2005). Also, neurons sensitive to movement did not appear to be clustered in the dorsolateral part of the STN, which is connected to motor and premotor cortical areas, and corresponds to the « motor » part of the STN. Although the small number of neurons sampled did not allow us to state whether the present findings are representative of STN recordings in general, the presence of neurons sensitive to expectation of reward in the ventral part of the STN is consistent with the findings of Matsumura et al. (1992).

### Functional significance of reward-related activity in STN

Although our findings add to the growing body of literature highlighting the role of the STN in motivation, it is not yet clear how reward signals found in the STN are used to influence behavioral output. One could speculate that changes in STN neuronal activity related to reward expectation or to the detection of the reward itself contribute to behaviors directed at obtaining the reward by maintaining the representation of an expected outcome and by monitoring the positive feedback which may serve to shape future behavior. An important feature of the reward signals generated by STN neurons in an instrumental task was their frequent combination with signals related to the movement. These combined changes in STN firing may reflect a mechanism linking the generation of motor behavior with the rewarding outcome. The specific function of STN in reward-guided behavior thus appears to be complex and further investigations of changes in STN neuronal activity employing appropriately designed tasks are needed to understand how this nucleus contribute to motor and reward processing in the basal ganglia circuitry.

Recently, the STN has been considered to be a key component of the brain network which mediates behavioral inhibition, particularly under circumstances requiring the active suppression of inadequate movements when several conflicting actions compete (Frank et al., 2007). At the moment, it is unclear how this conception can be matched with reward signals generated by STN neurons such as those described here. However, even seemingly simple motor behaviors consist of a spatiotemporal organization of distinct motor components, and STN neurons may intervene to facilitate or suppress these components to complete the whole behavior. In this regard, combined increases and decreases in STN activity may reflect a mechanism that releases from inhibition actions and maintains inhibitory control over others which is crucial for reward-oriented behavior.

## Conclusion

In summary, the data gathered in the present study provide further evidence for a contribution of the STN to motivational control of behavior in the non-human primate. Detailed understanding of the specificity of reward processing within single STN neurons could lead to a better appreciation of the influence of this nucleus on motivated behaviors under normal and pathological conditions in humans. This is particularly relevant in view of the current interest for surgical therapy aimed at treating psychiatric disorders associated with impaired reward and motivational processes, including drug addiction.

## Author contributions

Juan-Francisco Espinosa-Parrilla and Paul Apicella designed the study, collected the data, and performed the analyses. Paul Apicella wrote the manuscript with the help of Christelle Baunez.

## Conflict of interest statement

The authors declare that the research was conducted in the absence of any commercial or financial relationships that could be construed as a potential conflict of interest.
